# Assessment of Vessel Density on Non-Contrast Computed Tomography to Detect Basilar Artery Occlusion

**DOI:** 10.5811/westjem.2019.12.45247

**Published:** 2020-04-13

**Authors:** Andrew W. Asimos, D. Richard Sassano, Stephen C. Jackson, Jonathan D. Clemente, Jeremy B. Rhoten, Brenda Chang, Michael S. Runyon

**Affiliations:** * Atrium Health’s Carolinas Medical Center, Department of Emergency Medicine, Charlotte, North Carolina; † Brody School of Medicine at East Carolina University, Greenville, North Carolina; ‡ Atrium Health’s Carolinas Medical Center, Department of Radiology, Charlotte, North Carolina; § Atrium Health’s Carolinas Medical Center, Department of Neurosciences, Charlotte, North Carolina; ¶ Atrium Health, Information and Analytics Services, Charlotte, North Carolina

## Abstract

**Introduction:**

Basilar artery occlusion (BAO) may be clinically occult due to variable and non-specific symptomatology. We evaluated the qualitative and quantitative determination of a hyperdense basilar artery (HDBA) on non-contrast computed tomography (NCCT) brain for the diagnosis of BAO.

**Methods:**

We conducted a case control study of patients with confirmed acute BAO vs a control group of suspected acute stroke patients without BAO. Two EM attending physicians, one third-year EM resident, and one medical student performed qualitative and quantitative assessments for the presence of a HDBA on axial NCCT images. Our primary outcome measures were sensitivity and specificity for BAO. Our secondary outcomes were inter-rater and intra-rater reliability of the qualitative and quantitative assessments.

**Results:**

We included 60 BAO and 65 control patients in our analysis. Qualitative assessment of the hyperdense basilar artery sign was poorly sensitive (54%–72%) and specific (55%–89%). Quantitative measurement improved the specificity of hyperdense basilar artery assessment for diagnosing BAO, with a threshold of 61.0–63.8 Hounsfield units demonstrating relatively high specificity of 85%–94%. There was moderate inter-rater agreement for the qualitative assessment of HDBA (Fleiss’ kappa statistic 0.508, 95% confidence interval: 0.435–0.581). Agreement improved for quantitative assessments, but still fell in the moderate range (Shrout-Fleiss intraclass correlation coefficient: 0.635). Intra-rater reliability for the quantitative assessments of the two attending physician reviewers demonstrated substantial consistency.

**Conclusion:**

Our results highlight the importance of carefully examining basilar artery density when interpreting the NCCT of patients with altered consciousness or other signs and symptoms concerning for an acute basilar artery occlusion. If the Hounsfield unit density of the basilar artery exceeds 61 Hounsfield units, BAO should be highly suspected.

## INTRODUCTION

For emergency physicians (EP), acute basilar artery occlusion (BAO) is an easily missed, devastating type of ischemic stroke. Since BAO results in brainstem, cerebellar, or thalamic compromise, patients can initially present with a broad range of signs and symptoms, including headache, neck pain, visual symptoms, vertigo, nausea, vomiting, altered consciousness, and impaired gait.^1^–^4^ Frequently, this progresses to serious sequelae, including seizures, tetraplegia, locked-in syndrome, or death.^5^ BAO has the highest mortality rate of all types of ischemic stroke,^6^ with a morbidity and mortality rate of acute BAO of 80%–90% without active intervention.^7^ The era of mechanical thrombectomy has considerably elevated the importance of early identification of acute BAO.^8^ A recent meta-analysis examining the relationship between recanalization of acute BAO and clinical outcomes from 45 studies (n = 2056), reported a twofold and 1.5-fold reduction in mortality and risk of death or dependency, respectively.^9^ Furthermore, outcome after BAO is largely dependent on early recanalization, highlighting the importance of early detection.^10^–^13^

While computed tomography angiography (CTA) is increasingly being performed as part of the initial imaging of patients suspected to have acute ischemic strokes involving the anterior circulation of the brain, due to the varied presentation of BAO, non-contrast computed tomography (NCCT) of the brain is often the only initial imaging modality obtained for these patients. Additionally, the interpreting radiologist is often not provided enough clinical information to consider and carefully evaluate for a BAO, so scrutiny is rarely applied by radiologists in assessing the basilar artery. While the hyperdense middle cerebral artery (MCA) sign has been studied extensively and may be identified in 35–67% of patients with clinical MCA stroke,^14^–^17^ a “hyperdense basilar artery sign” (HDBA) is considerably less established. Reasons for this include concerns that posterior fossa artifact may alter the vessel density and the lack of a comparable (paired) artery to evaluate for asymmetry. Furthermore, the ability of EPs to assess the presence of a HDBA has never been reported. If EPs can reliably assess for a BAO by identifying an HDBA with sufficient accuracy to help guide immediate performance of a confirmatory CTA, this could lead to earlier identification of BAO, especially when the initial interpreting radiologist overlooks an HDBA. This has the potential to dramatically affect the outcome for some patients eligible for mechanical thrombectomy.

We aimed to do the following: 1) investigate the diagnostic accuracy and agreement of qualitative assessment of the HDBA sign on NCCT by EPs and a novice reader in patients with a CTA or digital subtraction angiography (DSA)-proven BAO; and 2) determine whether the quantitative Hounsfield unit (HU) BA density measured by EPs could increase accuracy and reliability over the qualitative assessment.

## METHODS

### Study Design

We conducted a case control study of patients with confirmed acute BAO vs a control group of acute stroke patients without BAO. Our institutional review board reviewed this study and determined it met criteria for waiver of authorization and expedited review.

### Study Setting and Population

We identified patients admitted to our comprehensive stroke center with a BAO by conducting a query of our institutional database (Premier Inc., Charlotte, NC) for the following *International Classification of Diseases*-9/10 codes during 2015–2017: I65.1, 433.00, 433.01, 433.20, 433.21, 433.30, 433.31, 433.90, I63.02, and I63.12. Additionally, we queried our stroke center’s database of suspected acute stroke patients to identify patients diagnosed with a stroke caused by an acute BAO. Patients identified in these queries were excluded if they did not have a BAO identified in a board-certified neuroradiologist’s imaging report of a CTA or DSA. Additionally, all CTA and DSA studies of BAOs were re-interpreted by a neuroradiologist (JDC) to confirm the presence of a total vs partial BAO. Occlusions were further classified as to whether they were limited to the basilar artery apex.

**Population Health Research Capsule**

What do we already know about this issue?

In patients with basilar artery occlusions, qualitative and quantitative assessment by radiologists of basilar artery density on computed tomography has been previously reported.

What was the research question?

To investigate the accuracy and agreement of these measurements by emergency physicians and a novice reader.

What was the major finding of the study?

A measurement above 61 Hounsfield units is highly specific for a basilar artery occlusion.

How does this improve population health?

These findings have the potential to improve the health of patients with basilar artery occlusion through earlier detection and treatment.

We excluded patients without an initial NCCT and confirmatory BAO identified on a CTA or DSA performed within 12 hours of the initial NCCT. Additionally, we excluded patients treated with systemic thrombolytics or mechanical thrombectomy between performance of the NCCT and CTA, MRA or DSA. This yielded 60 confirmed BAO patients. Our control patients consisted of 65 emergency department (ED)- suspected acute stroke patients randomly selected from our stroke center’s database (REDCap) who met the following criteria: performance of brain NCCT and CTA studies of both the head and neck within the time specifications described above and without an arterial occlusion or dissection identified on any CTA.

### Measurements

We randomly loaded the 4–5 millimeter (mm) NCCT axial imaging slice series of all patients into an imaging database. To assess intra-rater reliability, a subset of the BAO and control CTs were loaded twice. Four reviewers, consisting of two attending EPs, a third-year emergency medicine (EM) resident, and a medical student, performed the measurements described below. The two attending physician investigators have 26 and 16 years of experience as emergency practitioners. A medical student was chosen to participate to determine if a novice clinician could perform the measurements after the instruction described below. The medical student’s qualitative assessments were performed on all scans. His quantitative assessments were limited to only 38 BAO and 49 control patients, since he did not have access to the picture archiving and communication system (PACS) Hounsfield unit (HU) measurement software for all measurements, as some cases were added to the study sample after completion of the student’s summer research externship. All reviewers were blinded to the CTA/DSA images, the neuroradiologist’s CTA/DSA interpretation, and each other’s measurements. Reviewers were not provided with any patient history or exam findings.

Development and delivery of investigator instruction on assessing basilar artery density on NCCT was performed by AWA. At the outset of the trial, all clinicians performing measurements received a 30-minute tutorial of basilar artery anatomy, its identification on axial NCCT images, qualitative assessment for a segment of hyperdensity, and the quantitative measurement described below. In the authors’ opinions, once the relevant anatomy is understood, the basilar artery is readily identified as a midline vessel ventral to the medulla and pons on axial NCCT slices of those regions.

**Figure 1:**
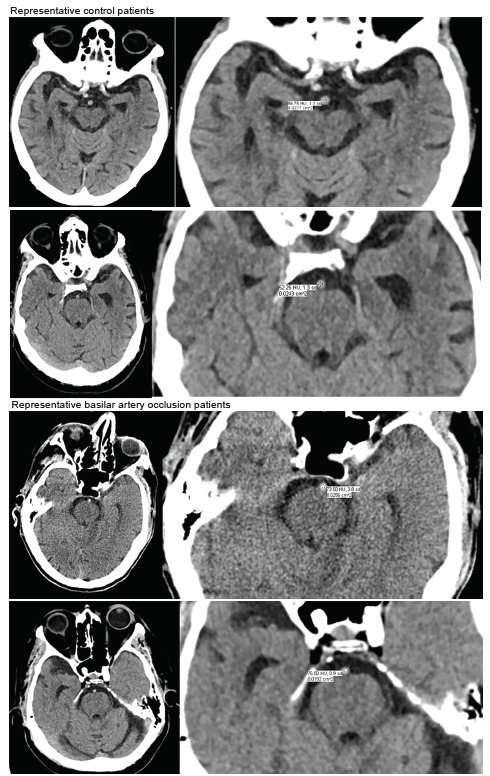
Representative control (above two patients, with full slice CT on left panel and close-up measuring Hounsfield units of basilar artery on right panel) vs basilar artery occlusion (bottom two patients).

First, each reviewer made a qualitative “yes/no” determination regarding the subjective presence of an HDBA based on scrolling through the axial slices covering the levels from the medulla to the midbrain. Next, on the axial slice in which the BA was determined to be most dense, the HU measurement of what was judged to be the BA artery was measured as follows: we zoomed the image to 400% and a circular region of interest measurement was obtained of the basilar artery density using Intellispace PACS, Enterprise Version 4.4.532.11 (Philips, N.V. Amsterdam, Netherlands). Each reviewer was instructed to exclude from the boundaries of the region of interest (ROI) any areas deemed to be beyond the margins of the artery and to limit the ROI circle circumference to the most homogeneously dense portion of the vessel possible (Figure 1). Additionally, we specifically asked each reviewer to document their qualitative assessment first to avoid any bias associated with the subsequent HU measurement.

All study measurements were entered directly by each reviewer into a REDCap database. Additionally, stroke program data abstractors, who were blinded to the basilar artery density measurements, extracted demographic and clinical characteristics for each patient from a prospectively maintained institutional database of patients who present to the ED with suspected acute stroke symptoms.

### Outcome Measures

Our primary outcome measures were sensitivity and specificity for BAO, based on the qualitative determination of the HDBA, with the aim to determine whether the quantitative HU BA density measured by EPs could increase accuracy over the qualitative assessment. Our secondary outcomes were intra-rater and inter-rater reliability of the qualitative and quantitative assessments.

### Data Analysis

Our reference standard for calculation of sensitivity and specificity was a CTA or DSA confirming partial or total BAO based on interpretation by a board-certified neuroradiologist. We used Fleiss’ kappa statistic to evaluate inter- and intra-rater reliability for the presence of HDBA as referenced against the interpretation of a neuroradiologist. The Shrout-Fleiss intraclass correlation coefficient (ICC 2,1) was used to evaluate single measures inter- and intra-rater reliability with absolute agreement and consistency of scores, respectively.^18^ We interpreted the categorical variable agreement according to the method of Landis and Koch.^19^ For the intraclass correlation coefficient (ICC) classifications, we used the categorization recommended by Koo and Li.^20^ We performed receiver operating characteristic (ROC) curve analysis to identify the optimal cut point of the HU BA lumen attenuation measurement associated with BAO presence. The optimal cut point was determined by maximizing sensitivity and specificity (Youden’s index).

## RESULTS

**Figure 2:**
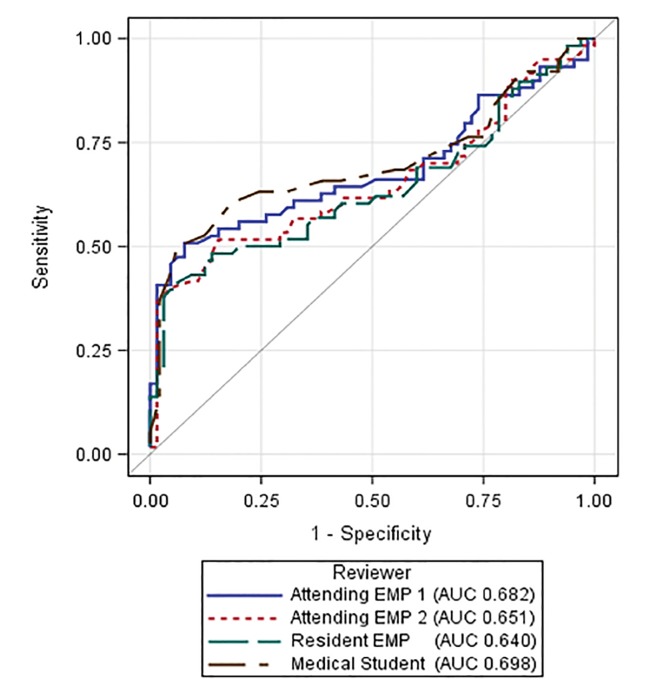
Receiver operating characteristics curve by reviewer for basilar artery density quantitative measurements. *EMP*, emergency physician; *AUC*, area under the curve.

Our analysis included 60 BAO patients and 65 control patients (Table 1). There were no statistically significant differences in demographic, clinical, or imaging characteristics between the two groups, other than the initial National Institutes of Health (NIH) Stroke Scale score. Among BAO patients, qualitative assessment for the HDBA sign performed inadequately to be of diagnostic utility in determining presence or absence of BAO on NCCT (Table 2). ROC curves for the quantitative measurements of each reviewer are shown in Figure 2. The data in Table 3 demonstrate that quantitative assessment improves the specificity of HDBA assessment for diagnosing BAO over subjective assessment. There was moderate inter-rater agreement for the qualitative assessment of HDBA presence (Fleiss’ kappa statistic 0.508, 95% confidence interval [CI], 0.435–0.581). Agreement improved for the quantitative assessments, but still fell in the moderate range (Shrout-Fleiss ICC 0.635, 95% CI, 0.624–0.982). Intra-rater consistency ranged from moderate to substantial among the four readers and improved to substantial for the two attending physician reviewers for the quantitative assessments (Table 4).

## DISCUSSION

Our study represents the first published assessment of HDBA by EPs and includes the largest number of BAO patients ever studied to evaluate HDBA. Since EPs routinely have greater knowledge of the clinical context prompting NCCT performance, and radiologists rarely comment qualitatively or quantitatively on the BA density, it is important to understand the proficiency, reliability, and accuracy of emergency medicine (EM) providers in assessing for an HDBA. Furthermore, understanding the performance of qualitative assessment alone vs quantitative assessment is essential. Our results indicate that subjective determination of HDBA by EM providers is insufficiently reliable and accurate.

However, quantitative HU measurement, which is a consistent capability of any digital imaging and communications in medicine reading software, at a consistent cutoff value above 61.0–63.8 greatly improved the specificity for identifying BAO. Moreover, our data suggest in a clinical scenario potentially consistent with a BAO, an HDBA with a HU measurement above 61 is highly specific for a BAO. This should prompt immediate consideration of this important and time-sensitive diagnosis. Nonetheless, despite the improved specificity, our results indicate that quantitative assessment of BA density on NCCT is insensitive for identifying a BAO.

Our findings differ somewhat from those found in other studies, which evaluated assessment of the HDBA by neuroradiologists and other experienced readers. In a study of three radiologists with varying degrees of neuroradiology expertise and overall experience, Connell et al reported similar sensitivities and specificities to ours for qualitative determinations for an HDBA.^21^ Alternatively, in two studies of neuroradiologist readers, the sensitivity of visual detection of the HDBA on NCCT was higher (81% and 71%^22^–^23^), as was the specificity (91% and 98%).^22^–^23^ Importantly, in both studies the image readers were aware that all patients were imaged for a possible BAO or other posterior circulation stroke, which most likely elevated pretest probability compared with our study.

Additionally, the studies performed by Ernst et al, Connell et al, and a much earlier and smaller study by Vonofakos et al,^24^ found the highest accuracy cutoff value for detection of BAO to be lower than that found in our study (40.0–46.5 HU and 61.0–63.8, respectively). Differing scanning parameters, newer generation scanning technology, or software differences in obtaining the HU measurements may explain the difference in the higher cutoff found in our study. Also, we relied on a more standard routine head CT slice thickness of 4–5 mm, while the other three studies used thicknesses down to 2 mm.

Despite the improved diagnostic specificity that quantitative measurements of a perceived HDBA may provide, NCCT is still a method that delivers an unacceptable percentage of false negative results in the diagnostic work-up of BAO. Furthermore, because of the high morbidity and mortality of untreated BAO, and because of its variable and fluctuating clinical presentation, obtaining a CTA, MRA, or DSA is essential when BAO is in the differential diagnosis. Nonetheless, we recognize that frequently only NCCT will be initially performed on many BAO patients.

BAO patients include those who present with the so-called “five Ds” of posterior circulation stroke, which include dizziness, diplopia, dysarthria, dysphagia, and dystaxia. Additionally, we recognize that in many of the most compelling presentations of BAO, such as altered consciousness, NCCT may only initially be performed, since intracerebral, subarachnoid, or intraventricular hemorrhage is frequently suspected. Our results suggest when those are not found, performing a HU measurement of the BA is a worthwhile undertaking by the EP. If he or she lacks this ability, the radiologist can assist in measuring the BA density at its most dense axial slice. Furthermore, if the density is above 61 HU in a clinical scenario concerning for a BAO, this devastating diagnosis should be considered and immediately pursued by performing a CTA in most cases.

Over 20 years ago, Perron and Kline introduced the “Blood Can Be Very Bad” mnemonic as a standardized method of cranial CT interpretation for EPs.^25^ “Blood” reminds the examiner to search for blood; “Can” prompts the examiner to identify four key cisterns; “Be” denotes the need to examine the brain; “Very” prompts a review of the four ventricles; and “Bad” reminds the examiner to evaluate the bones of the cranium. We propose that mnemonic be expanded to “Blood Can Be Very, Very Bad” to additionally remind EM practitioners to assess the density of “vessels,” not only qualitatively but also quantitatively in the appropriate scenarios. Additionally, our reliability data suggest that in the context of teaching this structured approach to reviewing a cranial CT, EM trainees may benefit from modest education in locating and assessing the basilar artery on CT.

## LIMITATIONS

CT scanners used were produced by several different manufacturers with inconsistent machine characteristics, which may have impacted the consistency of basilar artery density and HU measurements obtained for both BAO and control patients.^26^ However, as demonstrated in Table 1, CT scanner characteristics were similar between both patient groups, and the majority of all patients had a voltage of 120 kilovoltage peak and similar mean and median tube currents. Control patients were randomly selected based on the criteria described in the methods, but were not matched based on clinical or imaging characteristics. Nonetheless, as detailed in Table 1, BAO and control patients had similar demographic, clinical, and imaging characteristics.

The interpreting EPs and student were unaware of the patients’ symptoms and signs. However, we purposefully chose this study design, as the clinical presentation of patients with BAO can be broad. This correlates to an initial assessment of many BAO patients that only includes a NCCT with thick slices reported, rather than performance of an NCCT with thin slices and inclusion of an intracranial CTA. Especially since some of our patients had only partial BAOs, or had occlusions limited to the apex of the basilar artery, we recognize that CTs with thinner slices may be more likely to demonstrate an HDBA in the setting of a BAO. However, we believe that our methodology of including all patients with any degree of BAO and examining for HDBA on 4–5 mm NCCT slices represents a relatively conservative, “real-world” investigation of the clinical utility of qualitative and quantitative assessment of the HDBA sign.

Finally, none of the CT interpreters in our study received formal neuroradiological training. It is unclear what amount of training would be needed by average EM practitioners to identify and measure basilar artery density reliably, but our data suggest that at least some minimal training would be required by EM trainees to perform the assessments described in this work.

## CONCLUSION

Our results highlight the importance of carefully examining basilar artery density when the NCCT does not show a hemorrhage or other obvious intracranial abnormality to explain altered consciousness or other signs and symptoms potentially referable to the posterior cranial circulation. Furthermore, if the HU density of the BA exceeds 61 HU, BAO should be highly considered. With the rapid progress of artificial intelligence and machine learning in CT interpretation, we anticipate the presence of an HDBA may be identified by a computer in the future. Until such an advancement is integrated into clinical practice, EM practitioners should consider quantitatively assessing the basilar artery in the appropriate clinical scenarios.

## Figures and Tables

**Table 1:** Demographic, clinical, and computed tomography (CT) characteristics.

	Occlusion (n=60)				Control (n=65)				

	N	%	Mean	Std Dev	N	%	Mean	Std Dev	P-value
Demographic characteristics									
Age	60	100	66.6	14.7	65	100	64.4	16.6	0.429
Median (IQR)			68	(57–78)			66	(51–77)	
Gender									0.061
Male	35	58.3			27	41.5			
Female	25	41.7			38	58.5			
Race									0.760
Caucasian	43	71.7			43	66.2			
African American	13	21.7			17	26.2			
Asian	2	3.3			1	1.5			
Missing	2	3.3			3	4.6			
Ethnicity									0.236
Hispanic	1	1.7			1	1.5			
Non-Hispanic	33	55.0			47	72.3			
Missing	26	43.3			17	26.2			
Clinical characteristics									
Initial NIHSS	41	68.3	13.2	10.3	62	95.4	2.7	4.1	<0.001
Median (IQR)			10.5	(4–22)			1	(0–4)	
Hypertension	50	83.3			49	75.4			0.274
Diabetes	16	26.7			25	38.5			0.161
Hyperlipidemia	29	48.3			36	55.4			0.677
Atrial fibrillation	14	23.3			10	15.4			0.260
Smoking	17	28.3			17	26.2			0.784
Hematocrit	60	100	42.2	5.1	65	100	40.5	6.6	0.199
Median (IQR)			211	(173–257)			229	(186–264)	
Platelet count	60	100	226	77	65	100	237	73	0.444
Median (IQR)			211	(173–257)			229	(186–264)	
Type of basilar occlusion									
Total	24	40.0			0	0			
Partial	19	31.7			0	0			
Total (Apex)	10	16.7			0	0			
Partial (Apex)	7	11.7			0	0			
CT scanning specifications									
Number of detectors									0.057
≥64 detectors	40	66.7			53	81.5			
<64 detectors	20	33.3			12	18.5			
Voltage (kilovoltage peak [kVp])									0.633
80	3	5.0			3	4.6			
100	1	1.7			0	0.0			
120	56	93.3			62	95.4			
Current (millampere-seconds [mAs])	60	100	261	111	65	100	284	57	0.133
Median (IQR)			263	(168–322)			280	(264–314)	

*Std Dev*, standard deviation; *IQR*, interquartile range; *NIHSS*, National Institutes of Health Stroke Scale. Comparisons between the occlusion and control group for categorical variables (gender, race, ethnicity, comorbidities, etc.) and continuous variables were made using chi-square tests and two-sample independent t-tests, respectively. Comparisons for voltage were made using the Kruskal-Wallis test.

**Table 2:** Subjective assessment of hyperdense basilar artery (HDBA) presence with basilar artery occlusion.

Reviewer	Sensitivity (95% CI)	Specificity (95% CI)
Attending EP 1	0.54 (0.41–0.67)	0.89 (0.79–0.96)
Attending EP 2	0.63 (0.50–0.75)	0.60 (0.47–0.72)
Resident EP	0.63 (0.49–0.75)	0.55 (0.43–0.68)
Medical Student	0.72 (0.59–0.83)	0.69 (0.57–0.80)

*CI*, confidence interval; *EP*, emergency physician.

**Table 3:** Optimal cut points for basilar artery density measurement (HU) of basilar artery occlusion.

Reviewer	Cut point	Sensitivity (95% CI)	Specificity (95% CI)	Area under the curve (95% CI)*
Attending EP 1	61.8	0.51 (0.37–0.64)	0.92 (0.85–0.98)	0.68 (0.58–0.78)
Attending EP 2	61.3	0.52 (0.38–0.63)	0.85 (0.75–0.94)	0.65 (0.55–0.75)
Resident EP	63.8	0.41 (0.29–0.55)	0.94 (0.88–0.98)	0.64 (0.54–0.74)
Medical Student	61.0	0.50 (0.34–0.66)	0.94 (0.65–1.00)	0.70 (0.58–0.82)

*HU*, Hounsfield unit; *EP*, emergency physician; *CI*, confidence interval

* 95% confidence intervals for the sensitivity and specificity at optimal cut points were bootstrapped with 2000 replicates, while the 95% confidence intervals for the area under the curve were obtained using DeLong’s method.

**Table 4:** Intra-rater consistency of qualitative and quantitative assessments.*

	Qualitative assessment of HDBA presence: Fleiss’ kappa statistic (95% CI)	Quantitative Measurement of Basilar Artery Density (HU): Shrout-Fleiss ICC – Consistency (95% CI)
Attending EP 1	0.85 (0.69–1.02)	0.90 (0.84–0.94)
Attending EP 2	0.67 (0.43–0.91)	0.85 (0.76–0.91)
Resident EP	0.71 (0.48–0.93)	0.38 (0.15–0.58)
Medical Student	0.67 (0.44–0.90)	

*EP*, emergency physician; *HDBA*, hyperdense basilar artery; *HU*, Hounsfield unit; *ICC*, interclass correlation coefficient.

* The number of duplicate reads was 43, except for the resident who lacked one duplicate read for the quantitative measurements. The medical student performed 43 duplicate reads on only the qualitative assessments.
